# The use of finite mixture models to examine the serum 25(OH)D levels among Saudis

**DOI:** 10.1371/journal.pone.0260748

**Published:** 2021-11-30

**Authors:** Ibrahim Al-Sumaih, Michael Donnelly, Ciaran O’Neill

**Affiliations:** 1 Centre for Public Health, School of Medicine, Dentistry and Biomedical Sciences, Queen’s University Belfast, Belfast, United Kingdom; 2 Ministry of Health, Riyadh, Saudi Arabia; University of Dhaka, BANGLADESH

## Abstract

**Background:**

Recorded serum 25(OH)D in survey data varies with observed and unobserved respondent characteristics. The aim of this study was to expose latent population sub-groups and examine variation across groups regarding relationships between serum 25(OH)D and observable characteristics.

**Methods:**

This study explored the role of unobserved heterogeneity on associations between surveyed 25(OH)D and various factors using a sample (n = 2,641) extracted from the Saudi Health Interview Survey (2013). Linear regression and finite mixture models (FMM) were estimated and compared. The number of latent classes in the FMM was chosen based on BIC score.

**Result:**

Three latent classes were identified. Class I (39.82%), class II (41.03%), and class III (19.15%) with mean 25(OH)D levels of 22.79, 34.88, and 57.45 ng/ml respectively. Distinct patterns of associations with nutrition, behaviour and socio-demographic variables were recorded across classes that were not revealed in pooled linear regression.

**Conclusion:**

FMM has the potential to provide additional insights on the relationship between 25(OH)D levels and observable characteristics. It should be more widely considered as a method of investigation in this area.

## Introduction

Vitamin D deficiency has been associated with a range of conditions including osteopenia and osteoporosis [1]; increased risk of fractures [2]; muscle weakness [3] and (among expectant mothers) preeclampsia [4]. Given the high global prevalence of vitamin D deficiency [5] and its impact on health, it is unsurprising that the identification of factors which elevate the risk of deficiency should attract the interest of researchers. Factors that have been associated with increased risk of deficiency include pregnancy [6], skin colour [7], abstinence from direct sun exposure [8], lower educational achievement, smoking, physical inactivity, poverty, obesity and infrequent consumption of milk [7]. Differences in risk have also been related to particular genotypes [9].

Studies examining the causes and consequences of vitamin D deficiency, however, have seen the use of multiple thresholds to define deficiency [10]. In many instances this is understandable given thresholds may differ for specific outcomes (such as bone health as opposed to muscle weakness for example); or populations (expectant mothers as opposed to otherwise healthy adults, for example). Even when used with respect to the same group and outcomes, however, differences in the threshold used to define deficiency exist. For example, while some US studies [10] use a threshold of 20 ng/ml to define deficiency and others [7] 10 ng/ml as the concentration of serum 25-hydroxyvitamin D [25(OH)D] for otherwise healthy adults, in the UK others have suggested 12 ng/ml [11]. In this context in practical terms this leaves the researcher seeking to distinguish between groups in terms of risk exposure or to characterise membership of those groups in the invidious position of assessing the robustness of findings by testing multiple thresholds without a clear rationale for selecting one over the other.

An alternative approach is to measure 25(OH)D status as a continuous variable when assessing risk. Conceptually “deficiency” then exists on a spectrum rather than as a dichotomous variable at a defined threshold [12]. While this may appear more realistic (if perhaps more difficult to interpret) here too though the issue is not straightforward. Thus, while groups with different levels of risk based on measures of 25(OH)D can be identified and characterised based on observable characteristics such as age or education, for example, these characteristics may, depending on the variable in question, provide only approximations of an underlying concept of interest rather than definitive markers. Using this information to target groups for public health messages then becomes more challenging. For example, chronological age may be strongly related to biological age as will risks associated with vitamin D at particular levels among otherwise healthy adults. In this situation it may be relatively easy to identify those whose age places them at risk with a degree of clarity and suggest remedial action such as use of supplements. With respect to diet, however, access to 25(OH)D may be only roughly approximated by questions on food consumption contained in surveys because of a lack of detail in survey data as well as issues of recall and reporting bias on the part of the respondent. Thus, while identifying groups with low 25(OH)D may be possible, relating this to dietary habits and thus advice in respect of these may be more difficult. More generally the role of unobserved heterogeneity–where we do not have an observable counterpart for a concept of interest or may have a counterpart for whom the relationship with the latent concept varies across groups—may give rise to estimation issues [13, 14]. With respect to vitamin D, for example, and its relationship with diet or exposure to sunlight, unobserved attributes such as the strength or frequency of supplement use, the skin surface area exposed to sunlight, the time of day and year when skin is exposed (and thus ultra violet light exposure) and genetic preconditions that are not typically recorded in survey data on which empirical analyses are based may complicate associations [15–17]. In this context even using continuous measures of 25(OH)D to better reflect the existence of risk on a continuum could result in misleading advice because of the crudeness of the observable characteristics and heterogeneity in their relationships with unobserved counterparts. It follows that misleading advice could be given on how to interpret risk and trade-offs between these that characterise groups among whom risk might vary.

In this situation finite mixture models (FMM) may offer an opportunity to explore unobserved heterogeneity among subpopulations whose 25(OH)D level vary and for whom distinct relationships may exist with observable characteristics. FMM is a probabilistic model for representing the presence of subpopulations within an overall population, without the requirement of observable characteristics to identify the sub-population to which an individual observation belongs [18]. In finite mixture modelling, the observed data are assumed to belong to unobserved subpopulations called classes, and mixtures of probability densities or regression models are used to model the relationships within these and observable characteristics with the outcome of interest. After fitting the model, class membership probabilities can be predicted for each observation and used to interpret the class. In this context FMM provides a strategy that allows us to distinguish between subgroups that are otherwise unobserved either because of deficiencies in survey data (e.g. unrecorded dietary patterns), or because the complexity of underlying concept (risk) involves distinct patterns of relationships in observable attributes across sub-populations that cannot be distinguished with observed data. In this context, that is, it in principle allows us to identify subpopulations among whom levels of 25(OH)D vary (and with them health risks) as does the relationship with observable attributes. Further it allows us to characterise membership of those groups based on differences between them in terms of their characteristics and the probability of group membership.

In this paper we use the FMM approach to identify population subgroups and the distinct relationships for them between 25(OH)D levels and observable characteristics. We subsequently examine factors associated with the probability of subgroup membership.

The remainder of the paper is developed as follows: in the methods section we set out the data used in the study and the techniques used to analyse this, explicating the FMM approach. In the results section we describe the data and present the results of the FMM as well as the post hoc characterisation of sub-groups. The discussion relates our findings to previous studies and discusses the potential insights afforded by the FMM approach, as well as the limitations and recommendations from our study.

## Materials and methods

### Data source

Data were taken from the Saudi Health Information Survey (SHIS) 2013. The SHIS is a multistage survey of Saudi residents aged 15 years and older conducted between April and June 2013 [17, 19–22]. The population were stratified into 13 strata based on the administrative regions. Primary sampling units were adopted from the Census Bureau of the Kingdom of Saudi Arabia which divided Saudi households into small clusters consisting of a hundred and forty households on average. Primary sampling units were drawn proportional to their size randomly from the 13 administrative regions. Fourteen households from each primary sampling unit was randomly selected. Then, head of household was approached and a roster of occupants constructed. Potential participants age 15 years or above were randomly selected from the roster to participate in the survey.

The survey collects data on a range of socio-demographic characteristics such as age, gender, educational attainment, marital status and income. Self-reported dietary data are also collected allowing a reasonably comprehensive characterization of the individual to be constructed. Physical data on height, weight, waist circumference and blood pressure were measured during the interview visit. Participants were referred to local clinics where blood samples were taken from which measures including serum 25(OH)D levels cab be determined.

### Sample

In this study, we extracted data on 2,641 respondents for whom complete data were available with respect to age, sex, use of vitamin D supplements, daily sun exposure in minutes, consumption of dairy products including milk, laban (fermented milk), yogurt, labneh (condensed yogurt), and cheese. Consumption of fish and eggs were also extracted as potentially rich sources of vitamin D. Other variables including current smoking status, (all self-reported) as well as measured BMI (used to define obesity status), waist circumference, HbA1c and systolic blood pressure. Precise details on the specification of each variable are contained in S1 Appendix.

The choice of variables was informed by the literature Liu et al. [7], for example linking 25(OH)D level with current smoking status, milk consumption and obesity, AlQuaiz et al. [23] linking low serum 25(OH)D with failure to use supplements, younger age, low milk intake and central obesity, Al-Daghri [24] linking 25(OH)D levels with sex, Kimlin et al. [25] linking it with sun exposure; Malandish et al. [26] linking it with physical exercise; Scragg et al. [27], with higher HbA1c and He and Scragg [28] among others with systolic blood pressure. Some of the variables were not included in the primary analysis because of potential collinearity, for example, dairy products other than milk along with milk. To avoid possible collinearity in the model, collinearity was checked using variance inflation factor. Variables were also subjected to transformations to examine the robustness of the result as can be seen in S2 Appendix.

### Statistical analysis

Descriptive statistics (mean, median and standard deviation for continuous variables, percentage for dichotomous variables) for the sample were estimated. We compared samples on which we had full data with those we excluded from the analysis because of missing data to explore possible selection bias. Two sample t-test were used to compare continuous variables. Chi-square test were used to compare categorical variables. The relationship between covariates and recorded 25(OH)D was explored using linear regression. Subsequently, the analysis was repeated using a finite mixture model approach.

In FMM an observed distribution is assumed to be comprised of a finite number of distinct unobserved distributions, one for each unobserved subgroup. The observed distribution *y*, that is, is assumed to come from *g* distinct classes f1, f2,…, fg in proportions π1, π2,…, πg.

The probability density function of a g-component mixture cab be written as:

$$f(y)=\sum_{i=1}^{g}\pi_{i}f_{i}(y|x\prime \beta i)$$

where π_i_ is the probability for membership the ith class—the probability of class membership ranging from zero to one and summing to one across classes -; fi(·) is the conditional probability density function for the observed response in the ith class model, x the vector of variables on which membership is conditioned on and β denotes the parameters of the distribution [29].

FMM uses the multinomial logistic distribution to estimate π_i_ as

$$\pi_{i}=\frac{\exp(\gamma_{i})}{\sum_{j=1}^{g}\exp(\gamma_{j})}$$

where γ is a linear prediction of the ith class [29].

Further discussion of the approach can be found in “Health Econometrics Using Stata” (Deb et al., 2017) [18] and Stata reference manual [29].

Here, the number of subgroups (latent classes) on which the model was estimated was increased iteratively starting with two. Consistent with the recommended approach in the literature we used the Bayesian information criterion (BIC) to determine the optimal number of class [18, 30]. The posterior probability of subgroup membership was estimated where a threshold of 0.5 was used to define membership–i.e. where an individual was more as opposed to less likely to be a member of a particular sub-group. Descriptive statistics for subgroups (mean or percentage in the case of continuous and dichotomous variables respectively) were estimated so as facilitate the characterization and comparison of subgroups. Sub-group membership defined categorically based on a probability of group membership at 0.5 or above was also examined in a logistic regression as a function of a range of variables to shed further light on the characteristics of sub-groups. The FMM approach has been used in such diverse areas as demand for medical care [31], disease risk [32] and perceived consumer risk [33] and its use is supported by popular software packages including Stata version 15 and later.

All analyses and data manipulations were undertaken in Stata version 15 (StataCorp LLC College Station, TX).

## Results

Approximately one quarter (2,641/10,735; 24.6%) of survey respondents were included in the study which represents 61.6% of participants for whom serum 25(OH)D data were available. A comparison of included and excluded participants did not indicate any significant differences in terms of sex (p value 0.6647), serum 25(OH)D (p value 0.1258), duration of sun exposure (p value 0.3467), consumption of vitamin D supplement (p value 0.7860), laban consumption (p value 0.8409), oily fish consumption (p value 0.1283), and egg consumption (p value 0.3075). Differences were observed with respect to BMI, waist circumference, HbA1c, blood pressure and consumption of other dairy products where the studied sample was higher in BMI (p value 0.0119), waist circumference (p value <0.0001), HbA1c (p value 0.0061), systolic blood pressure (p value 0.0101) and milk consumption (p value 0.0004); and lower in terms of consumption of yogurt (p value <0.0001), labneh (p value <0.0001), and cheese (p value 0.0012).

Table 1 presents descriptive statistics for the respondents included in the analyses. As can be seen, average serum 25(OH)D level is 34.49 ng/ml and ranges from 1.23 to 149.6 ng/ml. Milk, laban, and cheese are the most popular dairy products consumed and only 3.71% of the sample consumed vitamin D supplements.

**Table 1:** Descriptive statistics of the sample (n = 2,641).

Variables	Mean	Median
**Age (years)**	39.81 (17.21)	37
**Serum 25-hydroxyvitamin D (ng/ml)**	34.49 (19.61)	29.72
**Daily sun exposure (minutes)**	17.30 (36.24)	4.29
**HbA1c (%)**	5.97 (1.39)	5.57
**Average systolic blood pressure (mmHg)** ^**1**^	121.04 (17.55)	119.5
**Waist circumference (in centimetre)**	89.29 (21.40)	91
**Female waist circumference, cm**	87.23 (20.07)	89
**Male waist circumference, cm**	91.41 (22.49)	93
**BMI (kg/m** ^**2**^ **)**	29.01 (11.64)	27.78
**Milk consumption (days per week)**	3.97 (2.68)	4
**Laban consumption (days per week)** ^**1**^	3.06 (2.42)	3
**Yogurt consumption (days per week)** ^**1**^	2.04 (2.16)	2
**Labneh consumption (days per week)** ^**1**^	0.87 (1.56)	0
**Cheese consumption (days per week)** ^**1**^	3.59 (2.40)	3
**Fish consumption (days per week)** ^**1**^	0.86 (1.33)	0
**Egg consumption (days per week)** ^**1**^	2.65 (2.02)	2
	**Frequency**	**%**
**Male**	1,302	49.30
**On vitamin D supplement**	98	3.71
**Generalized obesity**	985	37.30
**Perform intense sport**	210	7.95
**Currently smoking**	252	9.54

^1^ missing values.

The ordinary least-squares (OLS) regression results–reported in Table 2 - show that 25(OH)D levels are positively related to age, sex (male), consumption of vitamin D supplements, milk and haemoglobin A1c (HbA1c). By contrast waist circumference is seen to be negatively related to 25(OH)D levels. Other variables included in the analysis were not significantly related to 25(OH)D levels.

**Table 2:** Results of linear regression coefficient and FMM classes (n = 2,641).

Variables	OLS		Class I		Class II		Class III	
	Coef. (95% CI)	P-value	Coef. (95% CI)	P-value	Coef. (95% CI)	P-value	Coef. (95% CI)	P-value
**Age**	0.25 (0.20,0.30)	<0.001	0.07 (0.3,0.11)	0.001	0.20 (0.04,0.36)	0.016	0.52 (0.36,0.68)	<0.001
**Male**	5.24 (3.56,6.91)	<0.001	3.66 (1.95,5.36)	<0.001	8.91 (6.30,11.53)	<0.001	0.02 (-6.24,6.27)	0.995
**Vitamin D supplement**	7.90 (2.88,12.93)	0.002	20.39 (16.26,24.52)	<0.001	-12.49 (-19.94,-5.04)	0.001	16.62 (4.01,29.23)	0.010
**Daily sun exposure**	0.006 (-0.01,0.02)	0.512	-0.01 (-0.02,0.03)	0.589	0.04 (0.01,0.07)	0.019	- 0.07 (-0.14,0.01)	0.097
**Milk consumption**	0.37 (0.11,0.64)	0.006	0.34 (0.15,0.54)	0.001	0.49 (0.11,0.87)	0.012	0.01 (-0.96,0.98)	0.984
**Waist circumference**	- 0.07 (-0.11,-0.02)	0.006	- 0.01 (-0.6,0.03)	0.607	- 0.07 (-0.12,-0.02)	0.009	- 0.16 (-0.30,-0.3)	0.017
**BMI**	0.01 (-0.11,0.14)	0.846	-0.03 (-0.14,0.09)	0.647	-0.04 (-0.09,0.02)	0.184	0.45 (0.22,0.68)	<0.001
**Current smoker**	-0.22 (-3.06,2.63)	0.882	- 0.19 (-2.36,1.98)	0.866	- 5.28 (-9.06,-1.51)	0.006	15.19 (0.67,29.72)	0.040
**HbA1c**	0.82 (0.26,1.38)	0.004	0.42 (-0.27,1.12)	0.233	1.04 (0.28,1.79)	0.007	0.15 (-2.11,2.40)	0.899
**Intense sport**	2.73 (-0.25,5.71)	0.073	0.45 (-1.62,2.52)	0.669	0.37 (-4.01,4.75)	0.867	12.96 (1.64,24.28)	0.025
**Constant**	20.50 (16.04,24.96)	<0.001	15.18 (26.53,65.35)	<0.001	21.68 (15.57,27.80)	<0.001	35.41 (18.43,52.38)	<0.001

Using the same group of independent variables, a three-class FMM model—chosen based on BIC—was estimated and also reported in Table 2. The FMM approach revealed distinct patterns of association between subgroups and covariates. Mean vitamin D levels varied across the subgroups, the mean concentration of serum 25(OH)D were 22.79, 34.88, and 57.45 ng/ml for class I, class II, and class III respectively. The percentages of the sample in each subgroup were 39.82, 41.03, and 19.15% for class I, class II, and class III respectively. The relationship between covariates and 25(OH)D levels varied markedly from those suggested in the OLS model. For example, while age remained a significant and positive predictor of 25(OH)D levels, the magnitude of the coefficient in class III was double that of class II and almost six times that of class I. Similarly, stark differences were evident with respect to sex and milk consumption which were significant in class I and II albeit differing in magnitude but non-significant in class III; by contrast in the OLS both were significant and positive. The coefficient on vitamin D supplements was positively significant and large in the case of class I and class III but negative in class II. Similarly, while sun exposure was significant and positive in class II, it had no effect in the other subgroups–again a subtlety concealed in the OLS analysis. BMI became positively significant in class III although waist circumference remains negative in class II and class III. HbA1c is positive and significant in class II but insignificant in the other classes. Performing intense sport is positive in class III. Status of currently smoking is negative in class II but became second largest positive in class III.

In Table 3 descriptive statistics for the three subgroups where posterior probabilities are used to assign individuals to particular subgroups are presented. As can be seen, compared to other classes, class I were younger, less likely to consume supplements and laban, more likely to consume cheese, had better control of HbA1c and blood pressure. Class II lay between the other two classes in terms of magnitude in most of the covariates except for sun exposure and consumption of laban. Class III were older, more likely to consume supplements, less likely to have sun exposure and had higher HbA1c and blood pressure.

**Table 3:** Descriptive statistics of posterior probability for 3 components FMM.

Variables	Class I		Class II		Class III	
	Mean	SD	Mean	SD	Mean	SD
**Age (years)**	38.58	16.88	40.20	17.13	44.99	18.25
**Sun exposure (minutes)**	16.44	35.81	19.94	39.60	13.12	23.45
**HbA1c (%)**	5.89	1.33	6.03	1.43	6.08	1.36
**Systolic blood pressure (mmHg)**	120.13	17.97	121.22	16.93	124.89	17.68
**Waist circumference (in centimetre)**	89.56	21.38	89.14	21.24	89.39	22.32
**BMI (Kg/m2)**	28.99	10.87	29.07	13.00	29.20	11.59
**Milk (consumption days)**	3.95	2.69	3.98	2.64	3.97	2.73
**Laban (consumption days)**	2.96	2.40	3.23	2.44	3.02	2.45
**Yogurt (consumption days)**	2.14	2.20	1.96	2.10	1.92	2.18
**Labneh (consumption days)**	0.91	1.58	0.88	1.61	0.68	1.29
**Cheese (consumption days)**	3.71	2.36	3.56	2.39	3.15	2.44
**Fish (consumption days)**	0.84	1.29	0.92	1.38	0.79	1.34
**Eggs (consumption days)**	2.71	2.07	2.57	1.96	2.63	1.96
	**Frequency**	**%**	**Frequency**	**%**	**Frequency**	**%**
**Male**	574	47.17	525	53.63	150	45.45
**Vitamin supplement**	38	3.12	38	3.88	21	6.36
**Generalized obesity**	458	37.63	354	36.16	130	39.39
**Intense sport**	99	8.13	76	7.76	25	7.58
**Current smoking**	125	10.27	84	8.58	39	11.82

The results of the logistic regression looking at associations with probability of class membership are reported in Table 4. The probability of being in class I is positively associated with yogurt consumption and negatively associated with being male, younger and laban consumption. Being a male, consuming more laban and fish, being a non-smoker, or longer duration of sun exposure was associated with a higher chance of belonging to class II. The chance of being in class III increased with age, being female, a smoker, taking vitamin D supplements, avoidance of sun or cheese consumption.

**Table 4:** Results of logistic regression coefficient of posterior probabilities for each FMM class (n = 2,578).

Variables	Class I		Class II		Class III	
	Coef. (95% CI)	P-value	Coef. (95% CI)	P-value	Coef. (95% CI)	P-value
**Age**	-0.01 (-0.01,-0.001)	0.026	0.00 (-0.01,0.01)	0.962	0.02 (0.01,0.03)	<0.001
**Male**	-0.22 (-0.40,-0.005)	0.015	0.35 (0.17,0.53)	<0.001	-0.29 (-0.56,-0.02)	0.036
**Laban**	-0.04 (-0.07,-0.005)	0.025	0.05 (0.02,0.09)	0.003	-0.003 (-0.06,0.05)	0.899
**Yogurt**	0.04 (0.002,0.08)	0.041	- 0.04 (-0.08,0.004)	0.076	-0.02 (-0.08,0.04)	0.478
**Cheese**	0.02 (-0.02,0.05)	0.313	0.003 (-0.03,0.04)	0.866	-0.07 (-0.12,-0.01)	0.019
**Daily sun exposure**	-0.001 (-0.003,0.001)	0.367	0.003 (0.00,0.004)	0.025	-0.004 (-0.01,-0.0001)	0.042
**Current smoker**	0.25 (-0.03,0.53)	0.082	-0.35 (-0.64,-0.05)	0.021	0.49 (0.08,0.89)	0.018
**Fish consumption**	-0.06 (-0.12,0.004)	0.066	0.07 (0.01,0.013)	0.032	-0.01 (-0.11,0.09)	0.794
**Vitamin D supplement**	-0.39 (-0.82,0.05)	0.085	0.16 (-0.28,0.59)	0.478	0.64 (0.13,1.15)	0.014

Notes: The result is adjusted for consumption of milk, labneh and egg, BMI, waist circumference, HbA1c and intense sport.

## Discussion

The FMM approach affords an opportunity to explore the existence of subgroups within an overall sample, without the requirement of delineating these based definitively on an observable characteristic or group of such characteristics. The opportunity to expose unobserved heterogeneity and explore different patterns of relationships with observable characteristics can afford insights that might otherwise be missed where, for example, coefficient estimates reflect averages across an entire population. Care is warranted in the interpretation of results, as is the case here, subgroups may occupy different parts of the distribution–in this case of 25(OH)D levels. Used carefully, the FMM method may nevertheless reveal relationships that might otherwise have been missed and provide valuable information to policy makers. With specific reference to this study, two key findings are noteworthy.

First, the study sheds light on the conflicting evidence regarding the role of specific variables in vitamin D levels. For example, while a substantive body of literature points to the existence of a relationship between sun exposure and 25(OH)D levels, our OLS regression and previous work using the same survey data failed to expose the existence of this relationship [22]. By contrast in studies of specific subgroups evidence for a relationship between sun exposure and 25(OH)D levels is mixed. Al-Daghri et al. [34], for example, in a study of adults and children found an association between 25(OH)D levels and sun exposure among dark skinned boys but not in other groups. Similarly, while Al-Raddadi et al. [35] in a study of Saudi adolescent females found no association between 25(OH)D levels and sun exposure, Alzaheb and Al-Amer [36] in a study of young adult Saudi females found inadequate sun exposure to be strongly related to vitamin D deficiency. In another study that examined determinants of serum 25(OH)D among a sample of African Americans and White American men based in Chicago, Murphy and colleagues found that sun exposure was not correlated to the serum 25(OH)D until the sample was partitioned based on ethnicity where sun exposure was found to be significant among White Americans only [37]. In our study, sun exposure was significant but only for class II, the group who had the highest mean levels of sun exposure and intermediate levels of 25(OH)D.

Similar findings are evident with respect to the role of vitamin D supplements. While our OLS results suggest use of supplements was a significant determinant across the entire sample, use being positively related to levels of 25(OH)D, the FMM results reveal that this relationship is magnified in class I and class III but negatively related to the serum 25(OH)D in class II. Elsewhere in the literature evidence is also mixed as to the role of supplements. While our OLS findings are supported by those of Tuffaha et al. [22] using the same survey, for example, and by AlQuaiz et al. [23] who, in a study of adult Saudis, found that among both males and females absence of supplement use was associated with low 25(OH)D levels, Kaddam et al. [38] found no relationship between use of vitamin supplements and vitamin D deficiency among younger Saudis (students) and a negative relationship among older Saudis (employees). These results serve as examples of the potential for unobserved heterogeneity to result in potential confusion. In our OLS results, for example, partitioning the sample along age and gender may, by creating subgroups, have increased the possibility of detecting relationships among these groups though sample size may have created issues here but other subgroups may have gone undetected while choices may have attracted allegations of data mining. Thus, our inability to control for skin pigmentation [24], use of the veil [39], air pollution (which might partially block UV exposure) [40] or use of sun screen [41] all of which remain unobserved could have served to mask relationships. This is, similarly, the case with respect to supplements where the strength and frequency of use remain unobserved.

This finding is of significance to the second key finding from the study. The potential for average effects estimated across pooled classes may misrepresent relationships for specific subgroups which carries with it the risk that misleading advice may be given to those seeking to determine an appropriate policy response. With respect to the role of supplements for example, our OLS results, supported by the findings of Tuffaha et al. [22], AlQuaiz et al. [23] and others could underpin advice to the public at large in Saudi Arabia to consume supplements. By contrast our results suggests that more nuanced advice with respect to the use of supplements, diet and sun exposure might be more appropriate. Supplements were important in explaining variations in 25(OH)D levels but not among all classes. Among those with the lowest level of 25(OH)D, its effects were particularly marked indicating potential therapeutic benefits of supplements. Among those in class II, they may be effectively a waste of money. More broadly, the importance of contextualising the results of any study with individual specific information seems clear.

A strong relationship between obesity and low serum 25(OH)D has been well established in the literature [42]. However, our study findings suggest a different relationship between serum 25(OH)D and types of obesity. The correlation between central obesity and 25(OH)D found in our study is supported by a recent meta-analysis by Hajhashemy et al. [43] which demonstrated a negative relationship between central obesity and serum 25(OH)D despite the high level of heterogeneity among the included studies. Similarly, another meta-analysis of observational studies, Rafiq and Jeppesen [44] confirmed the negative effect of BMI on 25(OH)D level. However, one of the included studies, Al-Elq et al. [45], was a cross-sectional study on 400 Saudi healthy adults which had found (among females) a positive correlation between generalized obesity and serum 25(OH)D which is similar to the correlation found in class III. The author failed to explain the unexpected correlation. Newly emerging literature provided us with possible explanations of the unexpected relationship between BMI and serum 25(OH)D seen in class III. Older adults may be more subject to spine deformity associated with decreasing bone mineral density which can result in loss of between 3 to 5 centimetres of their adulthood height, with more height loss occurring in women [46]. Thus, BMI could give a misleading impression of obesity measures (and benefits) especially among older women. Other explanations are of course also possible. Thus, BMI as a measure of adiposity lacks the ability to distinguish between the proportion of fat and the muscular tissue in the body especially in athletes, this potentially complicating the interpretation of results. Results from Al-Elq et al. was not exclusive as another cross-sectional study from China have reported similar correlation. Foo et al. have examined determinants of serum 25(OH)D among Chinese adolescent girls and found that BMI is positively related to serum 25(OH)D [47].

Smoking is a known risk factor for vitamin D deficiency for its various mechanism that affect vitamin D synthesis and metabolism [48, 49]. What is surprising is that smoking in class III was found to have positive correlation with serum vitamin D which warrants further investigation. The unexpected association between smoking and serum 25(OH)D could be explained by the former’s correlation with a variable excluded from our analysis and caution is warranted when interpreting this result. However, replacing smoking status with cheese consumption (as can be seen in S2 Appendix) also resulted in three classes in which class III shows another interesting negative correlation between serum 25(OH) levels and cheese consumption.

As well as imprecision in the measurement of consumption, variation in the effect of dairy products on serum vitamin D could be explained by the genetic or ethnicity factors. For example, compared to fortified milk, unfortified milk contains very little vitamin D and an adult would be required to drink more than five litres of milk daily to gain the daily required vitamin D [50]. The content of vitamin D in Saudi dairy products varies from very little to moderate levels [51]. The average milk consumption in our sample is 4 days per week and does not vary in subclasses which could be enough if the chosen milk products were optimally fortified especially with the increasing awareness of the beneficial implication of vitamin D among Saudi population [52]. Similarly individuals may have a genetic predisposition that affects their response to vitamin D supplements [53] or from other sources.

Different definitions of vitamin D deficiency and insufficiency lead to different prevalence rates and management protocols and add confusion to the correlations between vitamin D deficiency and other diseases [54]. Our study underscores the complex nature of relationships between observable population characteristics and 25(OH)D levels that must be negotiated in the interpretation of reported findings and the framing of public health messages based on those findings.

It is important to note a number of limitations in our study. First, our data is cross sectional in nature and while we find a number of interesting associations, we can make no claims as to causal relationships between levels of vitamin D and the independent variables used to explain variations in it. Were a panel data set available causal relationships might be more fully examined, including for example the impact of initiation of supplement use on vitamin D levels. Second, (and in some respects at the heart of our analysis) is that we have incomplete information on many aspects of lifestyle or other attributes that might affect risk such as genetics. Were it possible to include additional observable data into our analyses we may have produced different classes to those produced as well as relationships between class membership and independent variables. An interesting avenue for further research would to compare FMM models estimated using panel data where additional information on panel members–such as duration of diagnoses or changes in lifestyle could be included and compared with analyses where it was omitted. Third, and again as noted, many of our variables likely exhibit measurement errors, for example in respect of use of supplements. This will likely continue to bedevil secondary analyses of data though FMM may perhaps offer a way of incorporating its role in particular circumstances e.g. where drug adherence is self-reported.

## Conclusion

Our study highlights additional insights afforded by use of the FMM approach in examining variations in levels of vitamin D. We identified three distinct subgroups among whom the average level of vitamin D and the relationship between vitamin D and observable characteristics varied. While for all 3 classes age was positively associated with vitamin D levels the magnitude of the coefficient differed by a magnitude of 6 among them. With respect to other independent variables even more distinct patterns were evident; supplement use was significant and positive for class 1 and 3 but negative for class 2; smoking status was significant and positive for class 3 but negative and significant for class 2 while being insignificant for class 1. As is evident by comparison with linear regression analysis these distinctions are concealed in the pooled analysis. The FMM approach may thus shed light on apparently conflicting relationships previously reported in the literature that underscores the careful framing of public health messages if public health is to be improved in a cost-effective manner.

## Supporting information

S1 Appendix Variable specification. (DOCX)

S2 Appendix Results of alternative model. (DOCX)
