# Supplementary material for: The use of finite mixture models to examine the serum 25(OH)D levels among Saudis
Source: PLoS One. 2021 Nov 30;16(11):e0260748. doi: 10.1371/journal.pone.0260748 (PMC8631613; doi:10.1371/journal.pone.0260748)
Supplement: S1 Appendix — (DOCX) [file pone.0260748.s001.docx]

**S1 Appendix. Variables specification.**

Age is a continuous variable measured in years.

Male is a dichotomous variable where male is given 1 and female is given 0.

Serum vitamin D is a continuous variable of serum 25-hyrdoxyvitamin D measured in ng/ml.

Milk consumption is a counting variable of days of consumption of milk per week.

Laban consumption is a counting variable of days of consumption of laban per week.

Yogurt consumption is a counting variable of days of consumption of yogurt per week.

Labneh consumption is a counting variable of days of consumption of labneh per week.

Cheese consumption is a counting variable of days of consumption of cheese per week.

Fish consumption is a counting variable of days of consumption of oily fish per week.

Egg consumption is a counting variable of days of consumption of egg per week.

Vitamin D supplement consumption is a dichotomous variable where 1 indicate using of supplement and 0 otherwise.

Performing of intense sport is a dichotomous variable where 1 indicate regular performing of intense sport and 0 otherwise.

Current smoking is a dichotomous variable where 1 indicate status of currently smoker and 0 otherwise.

Waist circumference is a continuous variable measured in centimetres.

BMI is a continuous variable of body mass index measured in kilogram per meter squared.

Generalized obesity is a dichotomous variable where 1 indicate BMI more than 30 kg/m2 and 0 otherwise.

HbA1c is a continuous variable of the percentage of haemoglobin A1c.

Systolic blood pressure is a continuous variable of average of second and third systolic blood pressure measured in mmHg.
