# Supplementary material for: The use of finite mixture models to examine the serum 25(OH)D levels among Saudis
Source: PLoS One. 2021 Nov 30;16(11):e0260748. doi: 10.1371/journal.pone.0260748 (PMC8631613; doi:10.1371/journal.pone.0260748)
Supplement: S2 Appendix — (DOCX) [file pone.0260748.s002.docx]

**S2 Appendix. Results of alternative model**

| **Variables** | **OLS** | | **Class I** | | **Class II** | | **Class III** | |
| --- | --- | --- | --- | --- | --- | --- | --- | --- |
|  | **Coef. (95% CI)** | **P-value** | **Coef. (95% CI)** | **P-value** | **Coef. (95% CI)** | **P-value** | **Coef. (95% CI)** | **P-value** |
| **Age** | 0.24 (0.19,0.28) | <0.001 | 0.08 (0.3,0.12) | <0.001 | 0.20 (0.003,0.39) | 0.047 | 0.45 (0.28,0.63) | <0.001 |
| **Male** | 5.13 (3.58,6.67) | <0.001 | 3.39 (1.74,5.03) | <0.001 | 8.02 (5.53,10.50) | <0.001 | 1.96 (-4.25,8.18) | 0.535 |
| **Vitamin D supplement** | 7.98 (4.16,11.80) | <0.001 | -2.43 (-5.28,0.41) | 0.594 | 6.95 (2.10,11.80) | 0.005 | 17.58 (3.58,31.57) | 0.014 |
| **Daily sun exposure** | 0.006 (-0.01,0.03) | 0.583 | 0.01 (-0.01,0.02) | 0.594 | 0.04 (0.004,0.07) | 0.028 | - 0.07 (-0.16,0.02) | 0.144 |
| **Milk consumption** | 0.41 (0.14,0.69) | 0.003 | 0.27 (0.07,0.48) | 0.009 | 0.64 (0.24,1.04) | 0.002 | 0.09 (-0.95,1.12) | 0.867 |
| **Cheese consumption** | -0.43 (-0.74,-0.12) | 0.007 | 0.12 (-0.21,0.45) | 0.470 | - 0.28 (-0.76, -0.19) | 0.236 | - 1.48 (- 2.82, - 0.14) | 0.030 |
| **Waist circumference** | - 0.07 (-0.11, -0.03) | <0.001 | - 0.01 (-0.05,0.04) | 0.799 | - 0.08 (-0.13, -0.03) | 0.003 | - 0.15 (-0.30, -0.001) | 0.048 |
| **BMI** | 0.02 (-0.05,0.08) | 0.619 | -0.04 (-0.14,0.06) | 0.420 | -0.03 (-0.07,0.01) | 0.158 | 0.41 (0.19,0.63) | <0.001 |
| **HbA1c** | 0.81 (0.26,1.36) | 0.004 | 0.40 (-0.38,1.18) | 0.312 | 1.13 (0.37,1.89) | 0.004 | 0.26 (-2.19,2.70) | 0.836 |
| **Intense sport** | 2.82 (0.05,5.59) | 0.004 | 0.40 (-1.67,2.47) | 0.312 | 0.89 (-3.13,4.92) | 0.663 | 13.25 (- 0.41,26.92) | 0.057 |
| **Constant** | 22.58 (17.94,27.23) | <0.001 | 14.91 (10.89,18.94) | <0.001 | 22.40 (15.33,29.47) | <0.001 | 43.79 (22.32,65.26) | <0.001 |
